# Around the Hospitals

**Published:** 1894-03-24

**Authors:** 


### Around the Hospitals.

Notice to the Managers of Institutions.—Special space is now reserved for the insertion of notices of hospital meetings and festivals. The Editor requests that all notices may reach The Hospital Office, 428, Strand, at least one week before the date of each meeting.

**The Bucks General Infirmary.**—This Infirmary commences the year with a small balance in hand, a fact for congratulation, though the management are rightly not content with the fact that the balance is a smaller one than the previous year could boast of. It certainly is a matter of regret that annual subscriptions have fallen off. The in-patients numbered 306 and the out-patients 1,402.

**Newcastle Royal Infirmary.**—The principal feature at the annual meeting of this institution was the decision to acquire a site for a new infirmary. If it had been possible to extend the infirmary on the present site a new building would not have been contemplated. The position for the proposed infirmary will probably be a spot adjacent to the Leazes Park. It is a little further from the Central Station than the present one, but is near many large works, and a contemplated tramway will render it still more accessible. The annual general meeting was adjourned to allow of a larger attendance of workmen's representatives. The working classes contribute generously to the infirmary, and it is an excellent feature that such contributions should be on the increase. The same may be said of the Northumberland miners, contributors now to the extent of £550 per annum, which sum is an increase of £350 on the contributions of the previous year. Owing to the generous donation of the late Lady Armstrong, the accounts of the institution show a balance to the good, but as was remarked at the meeting, such occasional strokes of good fortune should not be calculated as regular income. If a larger infirmary is erected fresh efforts to secure further annual contributions will have to be made. The necessity of a new building is generally admitted, and some of the funds are already promised.

**Chester General Infirmary.**—This institution has shown a good deal of activity during the past year, both in the matter of an increased number of patients and in improvements. The whole of the sanitary arrangements have been reconstructed. To effect this £2,000 of stock had to be sold out, £1,372 being devoted to this purpose. Other improvements effected free of cost to the institution have been the reflooring of a ward by the Chester Yeomanry Cavalry in memory of their late brother officer, Colonel Scotland; new beds and furniture by a grateful patient and collections by Nurse Mary; and balconies in the front of the hospital presented by Colonel Mascie Taylor. The Duke of Westminster is a liberal supporter of the institution, and he devotes the proceeds of visitors' fees to Eaton to it. These amount to £500 or £600 yearly. During 1893 1,317 in-patients and 4,245 out-patients were treated at a cost of £45 15s. 7d. per occupied bed, a decrease of nearly £3 per bed on the previous year's expenses. The ordinary income amounted to £5,601, special receipts being £597. Several legacies were received and promised.

